# Decreased Circulating Levels of APRIL: Questioning Its Role in Diabetes

**DOI:** 10.1371/journal.pone.0140150

**Published:** 2015-10-15

**Authors:** Adriana Carvalho-Santos, Marcelo Ribeiro-Alves, Luciene Carvalho Cardoso-Weide, Joyce Nunes, Lia Rafaella Ballard Kuhnert, Analucia Rampazzo Xavier, Samuel Cunha, Michael Hahne, Déa Maria Serra Villa-Verde, Carla Eponina Carvalho-Pinto

**Affiliations:** 1 Laboratory on Thymus Research, Oswaldo Cruz Institute, Fiocruz, Manguinhos, Rio de Janeiro, Brazil; 2 HIV/AIDS Clinical Research Center, National Institute of Infectology, INI, Fiocruz, Manguinhos, Rio de Janeiro, Brazil; 3 Faculty of Medicine, Fluminense Federal University, UFF, Niterói, Rio de Janeiro, Brazil; 4 Laboratory of Experimental Pathology, Biology Institute, Fluminense Federal University, UFF, Niterói, Rio de Janeiro, Brazil; 5 Institute of Molecular Genetics of Montpellier, Montpellier, France; Universidade de Sao Paulo, BRAZIL

## Abstract

Diabetes *mellitus* is a chronic disease that affects over 382 million people worldwide. Type-1 Diabetes (T1D) is classified as an autoimmune disease that results from pancreatic β-cell destruction and insulin deficiency. Type-2 Diabetes (T2D) is characterized principally by insulin resistance in target tissues followed by decreased insulin production due to β-cell failure. It is challenging to identify immunological markers such as inflammatory molecules that are triggered in response to changes during the pathogenesis of diabetes. APRIL is an important member of the TNF family and has been linked to chronic inflammatory processes of various diseases since its discovery in 1998. Therefore, this study aimed to evaluate APRIL serum levels in T1D and T2D. For this, we used the ELISA assay to measure serum APRIL levels of 33 T1D and 30 T2D patients, and non-diabetic subjects as control group. Our data showed a decrease in serum APRIL levels in T1D patients when compared with healthy individuals. The same pattern was observed in the group of T2D patients when compared with the control. The decrease of serum APRIL levels in diabetic patients suggests that this cytokine has a role in T1D and T2D. Diabetes is already considered as an inflammatory condition with different cytokines being implicated in its physiopathology. Our data suggest that APRIL can be considered as a potential modulating cytokine in the inflammatory process of diabetes.

## Introduction

Diabetes *mellitus* is a chronic disease that affects over 382 million people worldwide and caused approximately 5.1 million deaths in 2013. The prevalence of the disease is growing in developing countries and almost half of the cases are undiagnosed. Thus prevention and management of diabetes complications such as cardiovascular disease is very challenging [1].

Type 1 Diabetes (T1D) is classified as an autoimmune disease that results from pancreatic β-cell destruction and insulin deficiency, while Type 2 Diabetes (T2D), the more prevalent form of the disease, is characterized mainly by insulin resistance in target tissues, followed by decreased insulin production due to β-cell failure [2]. Since its association to obesity and activation of immune cells, T2D has been considered as an inflammatory metabolic disorder characterized by decreased islet size and insulin production [3]. In chronic diseases, such as diabetes, low grade of inflammation occurs without infection [4]. In T1D pancreatic islet inflammation (insulitis) contributes to the progressive loss of insulin-producing β-cells [5]. It is challenging to identify immunological markers such as inflammatory molecules that are triggered in response to changes in the metabolism [6]. Among circulating pro-inflammatory cytokines, tumor necrosis factor-α (TNF-α) expression was shown to be increased in obese rodents, leading to decreased insulin signaling in target tissues [7]. Furthermore, obese human subjects showed an increase in TNF-α mRNA expression in adipose tissue which was reversed by weight loss with increase in insulin sensitivity [8]. Gene knockout or neutralization of TNF-α with antibodies improved glycemia in rodents [9], although TNF-α neutralization approaches are not possible in humans [10]. Since its discovery by Hahne and collaborators in 1998 [11], a wide range of APRIL (member of TNF family) functions have been suggested such as increase of B-cell survival, co-stimulation of B-cell proliferation and antigen presentation in B-cells. APRIL can be produced by monocytes, dendritic cells, macrophages, T cells, acting solely as a secreted factor [12]. It can interact with receptors, the transmembrane activator and calcium modulator cyclophilin ligand interactor (TACI) and B cell maturation antigen (BCMA). APRIL can also bind to heparan sulfate proteoglycans (HSPGs) [13, 14].

APRIL plays a regulatory function in humoral response and several lines of evidence suggest that this cytokine is important in the establishment and/or maintenance of autoimmune diseases including systemic lupus erythematosus (SLE); rheumatoid arthritis (RA), Hashimoto’s thyroiditis and diabetes [15–19]. This study was undertaken to evaluate the role of APRIL in T1D and T2D. We showed a decrease of serum APRIL levels in T1D and T2D patients, as compared to healthy individuals and that this decrease is slightly negatively correlated with fasting glucose. Our data suggest that the function of APRIL deserves further investigation, emphasizing the importance of identifying relevant biomarkers for detection of immunological events related to diabetes.

## Methodology

### Patients and controls

This study was approved by the local Ethics Committee of Medicine School, Fluminense Federal University (UFF), Hospital Universitário Antonio Pedro (HUAP), CAAE n° 0065.0.258.000–10. Voluntary informed consent was obtained from every human subject involved in the study. Informed consent was co-signed by the project coordinator and two witnesses. The consent provided information about the study, peripheral blood collection and applied laboratory technique. The cohort recruitment was based on the criteria of the Endocrinology Service of Medicine School (UFF) for diabetes classification (glycemia > 126 mg/dL). In addition, patient’s histories were extensively analyzed. Were included in the study 33 T1D and 30 T2D patients, as well as 57 non-diabetic subjects (men = 23, women = 34) used as a control group. The control criteria of inclusion in the study were: (i) Non-diabetic subjects or normal glucose homeostasis (glycemia < 99 mg/dL and Hb1Ac < 5.5% or 37mmol/mol). (*ii*) Age similarity (± 5 years) to diabetic subjects. Exclusion criteria: (*i*) patients with alterations in insulin levels or glucose homeostasis, (*ii*) subjects with viral and rheumatologic diseases, including malignancy or sepsis, (*iii*) pregnant women, (*iv)* subjects with positive anti-nuclear antibodies (ANA > 1:80) and high levels of C-reactive protein (CRP > 0.5 ng/mL).

### Determination of APRIL in serum

Serum samples obtained from patients and control subjects were stored at -20°C until use. Determination of APRIL levels was done using Human APRIL Platinum ELISA (eBioscience, Vienna, Austria) according to the manufacturer’s instructions. Briefly, an anti-human APRIL coating antibody was adsorbed onto microwells and bound to human APRIL present in the samples or standard. Serum samples were diluted (1:2) in sample diluent and the standard curve was constructed ranging from 50ng/mL to 0.78ng/mL (serial dilution: 1:2). Each sample, standard, blank and optimal control was used in duplicate. A biotin-conjugated anti-human APRIL antibody was added and bound to human APRIL captured by the first antibody. Following incubation, unbound biotin-conjugated antibody was removed during a wash step. Then, streptavidin-horseradish peroxidase (HRP) was added to interact with biotin-conjugated anti-human APRIL antibody and, after a wash step, TMB substrate solution (tetramethyl-benzidine) was added. A colored product was formed in proportion to the amount of human APRIL present in the samples or standard. The reaction was terminated by addition of 1M phosphoric acid and absorbance was measured at 450 nm. APRIL concentration was determined upon the standard curve.

### Statistical analysis

Whenever required, data were power transformed by selected one-parameter Box–Cox transformations (power transformations), and adherence tests (Kolmogorov-Smirnov test) were conducted for the assumption of normality distribution. Whenever required, dependent variables were corrected for confounders given by age and gender. Hypotheses test for means differences were conducted either by T-Student or Welch tests, after Levene’s test to assess the equality of variances between groups. Whether there were three groups, means were compared by one-way Analysis of Variance (one-way ANOVA) followed by pair-wise Tukey multiple comparisons of means with 95% family-wise confidence level. Bartlett’s test was conducted to assess homogeneity of variances among groups. Associations between paired dependent variables were assessed by Pearson's product-moment correlation. Results were represented in box and strip plot displaying each sample and the five number summary statistics for each group. Two-tailed levels of significance less than or equal to 0.01, 0.05 and 0.1 were considered as "highly significant", "significant" and “suggestive”, respectively. All analysis was conducted in the environment R 2014 [20].

## Results

### Patient’s characteristics

A total of 33 T1D and 30 T2D patients were included in this study, with the patient’s characteristics being depicted in Tables 1 and 2 respectively. The mean age for T1D patients was 19.94 ± 6.94 for women and 17.16 ± 6.58 for men (range 6 to 34 years). There was no significant difference between the groups. In relation to T2D patients, the mean age was 58.08 ± 12.00 for women and 54.00 ± 12.56 for men (range 24 to 80 years) so, the Mann-Whitney test showed p = 0.41. Tables 1 and 2 also display the values of fasting glucose (FG, mg/dL) and glycated hemoglobin (HbA1c % and mmol/mol) that were determined at the same time of serum collection. It is noteworthy that HbA1c is an important issue for clinical laboratories for monitoring diabetes control. It is generally considered as an accurate index of long-term blood glucose regulation. Moreover, it can be responsible for cardiovascular complications. According with *Diabetics Control and Complications Trial (DCCT)* the reference for serum HbA1c involves three situations: no diabetes (4 to 5.6%), increased risk of diabetes or pre-diabetic (5.7 to 6.4%) and diabetic (6.5% or higher) [1,3]. In our study, 65% of patients in T1D group and 77% of the T2D patients presented HbA1c levels above 9.0% and there was no significant difference between women and men. Furthermore, patients were under similar treatment conditions. T1D treatment involved the recombinant human insulin, NPH (67%); regular insulin (33%); synthetic rapid-acting insulin, NovoRapid (52%); and others: Humalog, Lantus, Levemir and Apidra (~10%). About 78% of patients used two or three types of insulin, and less than 10% use antihypertensive drugs. Considering T2D, 80% of patients were treated with a combination of insulin, antidiabetic or antithrombotic substances, inhibitors of cholesterol, like Sinvastatin and antihypertensive drugs.

**Table 1:** Clinical features of Type 1 Diabetes (T1D) patients.

Gender	n	Age±SD	FG±SD/n	HbA1c±SD/n	^#^HbA1c±SD/n	DD±SD/n
F	21	19.94±6.94/21	168.47±81.83/18	9.71±2.64/18	83±29/18	8.72±6.32/18
M	12	17.16±6.58/12	163.58±92.56/12	9.05±1.83/12	75±20/12	10.58±8.2/12

SD: standard deviation; FG: fasting glucose (mg/dL); HbA1c: glycated hemoglobin (%)

^#^HbA1c (mmol/mol, recommended units according to International Federation of Clinical Chemistry-IFCC); DD: disease duration (years)

**Table 2:** Clinical features of Type 2 Diabetes (T2D) patients.

Gender	n	Age±SD	FG±SD/n	HbA1c±SD/n	^#^HbA1c±SD/n	DD±SD/n
F	18	58.08±12.00/18	156.4±69.41/12	9.71±2.85/11	77±31/11	11.4±6.81/10
M	12	54.00±12.56/12	196.7±67.99/10	9.6±2.27/9	81±25/9	17.4±18.6/10

SD: standard deviation; FG: fasting glucose (mg/dL); HbA1c: glycated hemoglobin (%)

^#^HbA1c (mmol/mol, recommended units according to International Federation of Clinical Chemistry-IFCC); DD: disease duration (years)

### Decrease of APRIL levels in the serum of diabetic patients

**Fig 1:**
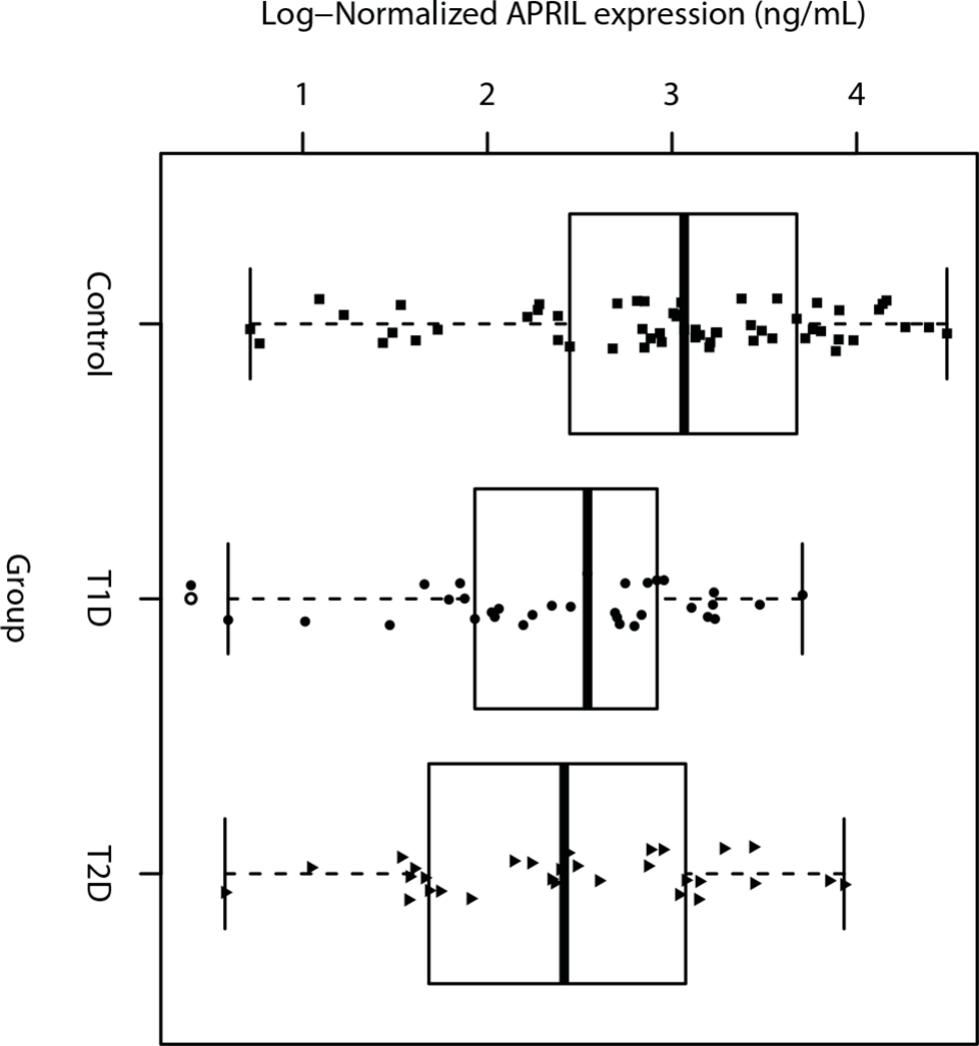
Circulating APRIL levels are decreased in diabetic patients. Log-Normalized (base = 2) APRIL serum levels (ng/mL) corrected by the square-root of age (confounder) of control (n = 57, squares), T1D (n = 33, circles), and T2D (n = 30; triangles) groups quantified by ELISA. We observed differences of -0.581 [-1.031; -0.132] (p-value = 0.007) and -0.540 [-1.003; -0.076] (p-value = 0.018) of means between groups T1D (2.39 ± 0.137) and control (2.97 ± 0.123), and between groups T2D (2.43 ± 0.151) and control, respectively.

Several lines of evidence suggest that APRIL is important to the establishment and/or maintenance of diseases that promote chronic inflammatory processes, including systemic lupus erythematosus (SLE), rheumatoid arthritis, Hashimoto thyroiditis [15, 17, 18]. To assess APRIL production in the inflammatory response of diabetes we measured the levels of circulating APRIL by ELISA. Our results showed that APRIL serum levels of T1D patients (n = 33; mean = 2.39 ± 0.137 ng/mL) were lower than those of healthy individuals (n = 57; mean = 2.97 ± 0.123 ng/mL), p = 0.007. The same response profile was observed in the group of T2D patients (n = 30; mean = 2.43 ± 0.151 ng/mL) when compared with the control group, p = 0.018 (Fig 1).

### Correlation between APRIL, fasting glucose and glycated hemoglobin

**Fig 2:**
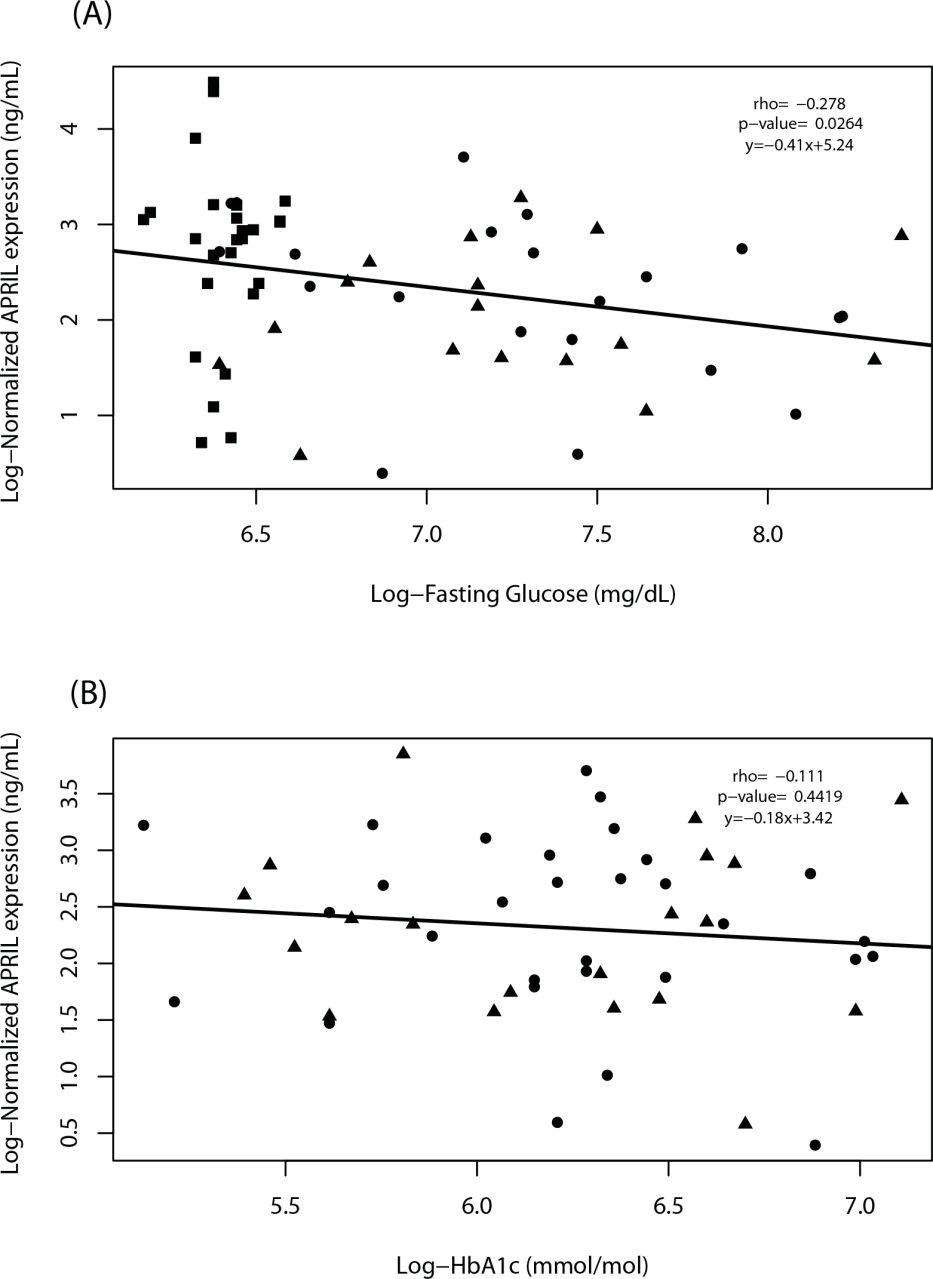
The fasting glucose (FG) levels are weakly negatively correlated with APRIL serum levels in diabetic patients. Log-Normalized (base = 2) APRIL serum levels (ng/mL) corrected by the square-root of age (confounder) and Log-Fasting Glucose mg/dL (base = 2) of control group (black squares; n = 57), T1D patients (black circles; n = 33), and T2D patients (black triangles; n = 30) quantified by ELISA (y-axis, **A**) are only weakly negatively correlated (rho = -0.278; p-value = 0.0264). Glycated hemoglobin (HbA1c) mmol/mol (base = 2) of the same groups (y-axis, **B**) are not correlated with APRIL serum levels (rho = -0.111; p-value = 0.4419).

The crucial criterion for diabetes classification used by the Endocrinology Service of Medicine School (UFF) is fasting glucose level. Our data indicated that serum APRIL levels in T1D and T2D patients were only weakly negatively correlated with fasting glucose (FG), rho = -0.278; p = 0.0264 (Fig 2A). No correlation was found between glycated hemoglobin (HbA1c) and APRIL serum levels rho = -0.111; p-value = 0.4419, in both T1D and T2D patients (Fig 2B). It is noteworthy that glycated hemoglobin is considered as an accurate index of long-term blood glucose regulation, and according with *Diabetics Control and Complications Trial* can be responsible for cardiovascular complications.

## Discussion

The role of inflammatory markers in chronic processes such as diabetes has been widely studied. T1D is well established as a multifactorial disease with an initial insulitis phase that involves dendritic cells, macrophages, B cells, CD4^+^ and CD8^+^ T cells. The next stage is a diabetic phase in which the onset of disease is caused by a progressive autoimmune destruction of insulin-secreting β-cells in the pancreas [3, 21]. In addition, autoantibodies have been shown to contribute as an important evidence of disease in humans and mice. They provide diagnostic and prognostic criteria, as markers for the course of disease [22]. The dialog between immune cells and the target β-cells during the course of insulitis is orchestrated by different cytokines and other inflammatory mediators. Among those, the TNF family member APRIL seems to be implicated in autoimmune response as a cytokine that regulates the homeostasis of B cells, among other functions. We have recently shown that APRIL acts as a negative regulator of collagen-induced arthritis (CIA), the most commonly used murine model for arthritis by regulating autoantibody production [16].

In this study, we observed a decrease of serum APRIL levels in T1D, as well as T2D patients, as compared to respective healthy control subjects. These data highlight the importance of better investigating how the pathogenesis of diabetes *mellitus* can be modulated by APRIL and its receptors. Mariño and collaborators [19] used the non-obese diabetic (NOD) mice as a model of T1D. They showed that blocking of the TNF family ligand members APRIL and B Cell Activating Factor (BAFF/Blys) in the pre-diabetic phase with a soluble decoy form of their common receptor (BCMA-Fc) resulted in prevention of autoimmune diabetes by reducing B cell numbers and decreasing autoimmune activity with the participation of T regulatory cells. On the other hand, previous reports in patients showed an increase of B Lymphocyte Stimulator (BLyS) serum levels in the chronic autoimmune disease SLE as compared to control subjects. No correlation between BLyS levels and disease activity was observed, whereas reports on serum APRIL levels in SLE were contradictory [15, 17]. Notably, APRIL-transgenic mice do not display clinical or immunological signs of spontaneous autoimmune disease. In fact, the immunomodulatory effect of APRIL in CIA is directly associated with the suppression of autoantibody production. This resembles the pathological importance of autoimmune complexes in human RA [15].

In relation to T2D, prospective studies have described elevated circulating levels of acute-phase proteins (such as C-reactive protein), haptoglobin, fibrinogen, serum amyloid A, as well as cytokines (such as TNF-α, IL-6 and IL-1β) and chemokines. These cytokines are crucial for acting in an autocrine and paracrine manner to promote tissue inflammation and insulin resistance [3, 6, 23]. In addition, other studies have discussed the mechanisms involved in the inflammatory response in T2D, such as hypoxia accompanied by accumulation of macrophages, adipocyte cell death, and recruitment of monocytes and lymphocytes [3, 24, 25]. APRIL can be secreted by inflammatory cells and their receptors may be expressed by lymphocytes [13].

A recent paper showed that patients with gestational diabetes *mellitus* (GDM), exhibited similar levels of APRIL in serum when compared to non-GDM individuals [26]. GDM, in addition to T1D and T2D, is an inflammatory condition and various cytokines have been implicated in its physiopathology [27].

Our data in T1D and T2D patients suggest that APRIL can be considered as a potential modulating cytokine in the inflammatory process of diabetes, emphasizing the importance of seizing new therapeutic windows as well as identifying relevant biomarkers for detection of immunological events related to the disease.
